# The functional consequences of Generalized Joint Hypermobility: a cross-sectional study

**DOI:** 10.1186/1471-2474-15-243

**Published:** 2014-07-21

**Authors:** Mark C Scheper, Janneke E de Vries, Birgit Juul-Kristensen, Frans Nollet, Raoul hh Engelbert

**Affiliations:** 1 Amsterdam School of Health Professions, Education of Physiotherapy, Amsterdam, The Netherlands; 2 Department of Rehabilitation, Academic Medical Center, University of Amsterdam, Amsterdam, The Netherlands; 3 University of Southern Denmark, Institute of Sports Science and Clinical Biomechanics, Research Unit for Musculoskeletal Function and Physiotherapy, Odense, Denmark

**Keywords:** Generalized Joint Hypermobility, Functional status, Dance, Pain

## Abstract

**Background:**

Generalized Joint Hypermobility (GJH) has been found to be associated with musculoskeletal complaints and disability. For others GJH is seen as a prerequisite in order to excel in certain sports like dance. However, it remains unclear what the role is of GJH in human performance. Therefore, the purpose of the study was to establish the association between GJH and functional status and to explore the contribution of physical fitness and musculoskeletal complaints to this association.

**Methods:**

A total of 72 female participants (mean age (SD; range): 19.6 (2.2; 17-24)) were recruited among students from the Amsterdam School of Health Professions (ASHP) (n = 36) and the Amsterdam School of Arts (ASA), Academy for dance and theater (n = 36) in Amsterdam, The Netherlands. From each participant the following data was collected: Functional status performance (self-reported Physical activity level) and capacity (walking distance and jumping capacity: side hop (SH) and square hop (SQH)), presence of GJH (Beighton score ≥4), muscle strength, musculoskeletal complaints (pain and fatigue) and demographic characteristics (age and BMI).

**Results:**

GJH was negatively associated with all capacity measures of functional status. Subjects with GJH had a reduced walking distance (B(SE):-75.5(10.5), p = <.0001) and jumping capacity (SH: B(SE):-10.10(5.0), p = .048, and SQH: B(SE):-11.2(5.1), p = .024) in comparison to subjects without GJH, when controlling for confounding: age, BMI and musculoskeletal complaints. In participants with GJH, functional status was not associated with performance measures.

**Conclusion:**

GJH was independently associated with lower walking and jumping capacity, potentially due to the compromised structural integrity of connective tissue. However, pain, fatigue and muscle strength were also important contributors to functional status.

## Background

An excessive range of motion, which is referred to as Generalized Joint Hypermobility, is present in in 2 to 57% of the child and adult population [1,2]. It is assumed that GJH is an expression of laxity of connective tissue, due to genetic alterations in elastic fibers and thus affecting the structural integrity of connective tissue throughout the whole human organism [3]. This in turn, affects the function of multiple organs and structures that are comprised of connective tissue.

Connective tissue laxity is also a clinical feature of specific pathological entities like Ehlers-Danlos Syndrome, Marfan or Ostegenesis Imperfecta [3]. These hereditary diseases of connective tissue (HDCT) are rare and can have serious consequences in terms of disability and in some cases may even result in death. In recent years the genetic origin of collagen diseases has been explored [4-7], whereas also a subgroup of connective tissue disorders was identified. This subgroup of connective tissue disorders shares the typical, although less severe, clinical presentation, but lacks biological markers [8]. These disorders are often referred to as Hypermobility Syndrome (HMS) or Ehlers-Danlos Syndrome (hypermobile type) [9]. The exact prevalence is unknown, and estimates do vary [10].

Not all individuals with GJH are symptomatic, some even take advantage of GJH and excel in certain sports like gymnastics, martial arts or dancing [11,12]. Others experience mild to severe musculoskeletal complaints [13], like joint pain in multiple joints [10], fatigue [13], diminished motor competence [14] and muscle weakness [15]. In a recent study by Scheper et al., GJH was also found to be independently associated with physical deconditioning, fatigue and psychological complaints in professional dancers who are considered to benefit from GJH in order to perform complex dance routines. These findings and other literature indicate that the consequences of GJH for performance remain poorly understood. If the assumption of connective tissue laxity is correct then GJH should also affect functional status, even in individuals who should excel in functional ability, like professional dancers.

Functional status is a multi-dimensional concept defined as a patient-oriented health outcome which contains aspects of individual daily functioning, including physical, psychological and social factors [16]. Functional status is often used as a primary outcome in a variety of study designs, whereas an operational definition is frequently lacking [17]. It can, however, be operationalized in both capacity and performance measures, where capacity refers to what a patient can do in a standardized environment, and performance to what a person does in daily life [18]. Although often reported by patients and clinicians, the evidence of impairments on functional status in symptomatic GJH is limited, as well as factors influencing functional status.

Weight bearing physical activities like standing, walking and participating in sports and leisure activities might be physically demanding in subjects with symptomatic GJH [19-22]. This is possibly due to the presence of pain, fatigue and reduced physical fitness that could negatively influence functional status [22-24]. However, how much these factors contribute to functional status remains unknown, as do the factors that would enable professional dancers to benefit from GJH instead of adding to functional disability.

Therefore, the aim of the present study was to investigate the association between GJH and functional status, in terms of capacity and performance. In addition the secondary objective was to explore the contribution of physical fitness and musculoskeletal complaints to this association.

## Methods

### Subjects

A convience sample of 72 female participants (mean age(SD); range: 19.6 (2.2; 17-24)) were recruited among students from the Amsterdam School of Health Professions (ASHP) (N = 36) and compared to age matched subjects from the Amsterdam School of Arts (ASA), Academy for dance and theater (N = 36) in Amsterdam, The Netherlands. Subjects recruited at ASA were students in their final year of professional dance education and were classified as professional dancers. Subjects were eligible for inclusion when orthopedic, cardio- pulmonary-, rheumatological-, neurological conditions or disorders influencing functional status were absent. Secondly, inclusion criteria implied absence of conditions or disorders that render the participant unable to understand the questionnaires or to adhere to the protocol. The study was designed according to the STROBE guidelines [25] (http://www.strobe-statement.org). Written informed consent was obtained from all participants according to the Declaration of Helsinki [26]. The study was approved by the medical ethical board of the Academic Medical Centre, Amsterdam, the Netherlands.

All outcome measures were classified according to domains of the International Classification of Functioning (ICF) [27], in order to provide a clear description of all outcome measures and their inter-relationship. The ICF is multidimensional model of functioning with activities and participation as the key construct. This model provides a framework to describe limitations associated with an individual’s functioning and identifies influencing environmental factors [27].

In the ICF domain body function and structure demographic data were collected regarding age, gender, height and weight. Standing height and weight were measured in a standardized method without wearing heavy clothing and shoes, and rounded to the nearest centimetre and 100 gram, respectively. Body Mass Index (BMI) was calculated with the formula weight/height^2^. All outcome measures were collected by two experienced physical therapists (MCS, JEV) and were assisted by four undergraduate physical therapists. In order to ensure uniformity, all assessors received an 8-week training regime. Data integrity was verified prior to the start of the study for all outcomes in terms of intra-rater reliability. Intra-rater reliability (ICC) varied between .81 and .94 and thus exceeding > .80, indicating excellent reliability.

### Generalized Joint Hypermobility

The presence of GJH was classified by the Beighton score, with a standardized and reproducible protocol [28]. The Beighton score consists of five clinical maneuvers performed bilaterally and scored dichotomously (0-1). A total score (ranging from 0 – 9) was derived by summation of all maneuvers: (1) passive opposition of the thumb to the flexor side of the forearm (shoulder 90° flexed, elbow extended and hand pronated), (2) passive dorsiflexion of the little finger >90° (elbow flexed 90°, the forearm and hand pronated resting on a table) (sitting), (3) passive hyperextension of the elbow >10° (shoulder 90° abducted and hand supinated), (4) passive hyperextension of the knee >10° (standing), (5) forward flexion of the trunk, with knees straight, so that the palms of the hands rest easily on the floor (standing). GJH was defined when a Beigthon score of ≥4 was obtained [29]. Beighton scores were determined by two assessors (MCS, JdV) independently in order to ensure the validity of the GJH classification, therefore inter-rater reliability was determined. Inter-rater reliability (ICC, (95% CI)) was .81 (.58 - .94). All visual Beighton observations were verified by the use of goniometer. In addition all subjects were screened independently (blind) by both assessors (MCS, JdV) on the bases of the Brighton criteria for HMS [29]. When the Brighton criteria were not fulfilled and GJH was accompanied by pain, symptomatic GJH was classified.

### Muscle strength

Muscle strength of the proximal and distal muscles in lower and upper extremities was measured bilaterally in a standardized way [30] with a hand-held dynamometer (Citec, Groningen, The Netherlands). Measurements were consecutively performed three times and the highest value was registered. In the upper extremity, shoulder abductors and grip strength were measured; in the lower extremity, hip flexors, knee extensors and dorsal extensors of the feet were measured. All measurements were performed according to the “break method” with the exception of the knee extension and grip strength. For these measurements the “make method” was applied due to the inability of the assessors to break the generated force of the participant [31]. Total muscle strength was calculated by a summation of all individual muscles (left and right) [31]. After each measurement subjects were asked if they were limited by pain, this was recorded.

### Musculoskeletal complaints

The extent of musculoskeletal complaints was assessed on two aspects: pain intensity and fatigue. Pain intensity was quantified according to the Visual Analog Scale (VAS) expressed in mm, ranging from no pain at all (score: 0 mm) to worst pain ever experienced (score: 100 mm) [23]. Subjects rated averaged pain intensity experienced in the last two weeks.

Fatigue was quantified by the Checklist Individual Strength (CIS) [32]. The CIS was designed to measure several aspects of fatigue: the subjective experience of fatigue, reduction in motivation, reduction in activity, and reduction in concentration. The CIS has been found reliable and valid in healthy controls, patients with chronic fatigue syndrome and other chronic diseases [32]. Total scores used for analysis were calculated through summation of all sub-items resulting in a score ranging from 0 (no fatigue) to 100 (very severe fatigue) [32].

### Functional status: performance

In the ICF domain activities, performance was defined as the execution of a task or action performed by an individual in real life situations, and is mostly quantified by self-reported properties.

For quantification of the self-reported performance, the Short Questionnaire to Assess Health enhancing physical activity (SQUASH) was used. The SQUASH instrument is designed to assess habitual activity level, and has been shown to be a reliable and valid questionnaire [33]. Data acquired from the SQUASH questionnaire were converted into Metabolic Equivalent Tasks (METS) for each individual domain (mobility, household, occupation and leisure time activities) according to the compendium of physical activities [34]. One MET equals the resting metabolic rate obtained during quiet sitting and equals an approximate oxygen uptake of 3.5 ml/kg/min. The oxygen expenditure for physical activities ranges, for example, from 0.9 MET for sleeping to 16 METS for running a 6 minutes mile. A total Physical Activity Level was calculated by summation of all individual domains. In addition subjects were questioned if this week was comparable to normal. In the case of a beyond average week, the differences from normal were registered and the Total PAL was adjusted accordingly.

### Functional status: capacity

Capacity is defined as the execution of a task or action performed by an individual in controlled, laboratory like conditions, and was quantified by the Six minute walk test (6MWT) and Jumping capacity tests. These outcomes were chosen derivatives of functional status as they only partly reflect real life activities.

The 6MWT was performed on an 8-meter track in a straight corridor as described by Gulmans et al. [35]. Participants were instructed to cover the largest possible distance in 6 minutes at a self-selected walking speed. Turns were made on both ends of the 8-meter track. The distance walked was recorded with a lap counter. Each time the patient returned to the starting line, the lap counter was clicked once. Every minute the patients were encouraged in a standardized way, and time was recorded with a stopwatch. At the end of the test, the patient was asked to stand still and the distance covered in the final partial lap was measured. Multiplying the number of laps by 16 meters and adding the additional meters of the final partial lap calculated the total distance. The 6MWT was found to be reliable and valid in order to quantify functional capacity [36].

In order to quantify jumping capacity the Side hop test (SH) and the Square Hop test (SQH) were performed [37]. For the SH, the subjects stood on the tested leg, with their hands behind their back, and jumped from side to side between two parallel strips of tape, placed 40 cm apart on the floor. The subjects were instructed to jump as many times as possible during a period of 30 seconds. For the SQH test, the subjects stood on the leg to be tested, with their hands behind their back, outside a 40 × 40 cm square marked with tape on the floor. A 10 cm frame was also marked around the square with tape. For the right leg, the subjects were instructed to jump clockwise in and out of the square as many times as possible during a period of 30 seconds. The number of successful jumps performed, without touching the taped frame, was recorded. Touching the taped frame was recorded as an error and, if more than 25% of the jumps had errors, a second trial of 30 s was performed after a 3-min rest period. For the left leg, the subject performed the test in a counter-clockwise mode. The total amount of jumps was used for analysis. After each measurement subjects were asked if they were limited by pain, this was recorded. Only one subject reported to be limited due to pain. When removing this subject from analysis, no effects of that individual was found on the regression models, therefore the subject was retained for analysis.

### Statistical analysis

Statistical analysis was performed in three stages: (1) Assessment of distribution and normality, (2) factor identification and (3) multivariate analysis. Firstly, the skewness of the data was assessed visually and by Kolmogorov-Smirnoff test. Normally distributed data were expressed by means and standard deviations (SD), whereas not normally distributed data were expressed as median (P50) and interquartile range (P25-P75).

Secondly, factor identification for multivariate analysis was performed by univariate analysis. In order to account for group specific differences (dancer versus non-dancer), differences were determined by an independent t-test. Variables with a p-value of <0.15 were retained for further analysis [38].

Finally, in order to investigate the association between functional status (dependent variables) and GJH (independent variable), controlled for potential confounding factors, a linear regression analysis was performed. Potential confounding factors were: age, group (dancers vs non-dancer), BMI, muscle strength, pain and fatigue. Results of linear regression are presented in regression coefficients/standard errors (B(SE)) with 95% confidence intervals (95%CI). All statistical analyses were performed in SPSS version 20.0. P-values <0.05 were considered to be statistically significant.

## Results

All initially invited subjects were willing to participate and all fulfilled the inclusion criteria and were included in the analysis (n = 72: 36/36). The presence of GJH was significantly higher amongst dancers (66%) in comparison to controls (29%) (X^2^ = 12.995, p = .001). An overview of the included study population is provided in Table 1.

**Table 1:** Subject characteristics distributed by recruitment location (non-dancer vs. dancer)

	**Non-dancer (n = 36)**		**Dancer (n = 36)**		***p-value***
Classified with GJH (Beigton ≥4: % positive)*	10 (29%) positive vs. 26 (61%) negative		24 (66%) positive vs. 12 (34%) negative		p = .004
	*Mean (SD)*	*Range*	*Mean (SD)*	*Range*	
Age (years)**	20 (2)	17.0–24.0	20 (2)	17.0–24.0	P = .999
**BMI (Kg/m**^**2**^**)**	**22.3 (2.5)**	**(18.5-28.4)**	**20.8 (1.8)**	**(16.8–23.5)**	**p = .002**
*Functional status: Performance*					
Total PAL (METS)	168.8 (94.4)	16.8–435.2	195.5 (104.4)	37.8–491.5	p = .234
*Functional status: Capacity*					
**6MWT (m)**	**575.3 (67.5)**	**418.5–704**	**630.8 (58.6)**	**492.0–780.0**	**p = <.0001**
**SQH (count)**	**98.6 (24.9)**	**50.0–144.0**	**117.2 (16.9)**	**67.0–150.0**	**p = <.0001**
**SH (count)**	**56.8 (20.1)**	**24.0-115.0**	**68.3 (21.1)**	**35.0–125.0**	**p = .015**
Muscle Strength (N)	2037.0 (337.2)	(1651.7–3313.3)	1988.6 (363.2)	(1387.7–2854.0)	p = .605
Pain intensity (mm)	43.5 (20.2)	(0.0–79.0)	38.3 (20.0)	(0.0–72.0)	p = .258
**Fatigue (CIS)**	**26 (21)**	**(5–97)**	**47 (17)**	**(10–83)**	**p = <.0001**

*Abbreviations:* Body Mass Index (BMI), Generalized Joint Hypermobility (GJH), Total Physical Activity level (PAL), Metabolic Equivalent Tasks (METS), Six minute walk test (6MWT), Square Hop (SQH), Side hop (SH), Newton (N), Checklist Individual Strength (CIS). *Chi-square analysis **age matched Significant associations are presented in bold.

### Univariate analyses

When accounting for differences between dancers and non-dancers (Table 1), dancers showed lower scores on BMI (T = 3.13, p = .002), higher scores on all capacity measures of functional status: 6MWT (T = 3.93, p = <.0001), SQH (T = 3.91, p = <.0001), SH (T = 2.49, p = <.015) and experienced increased fatigue (T = 4.78, p = <.0001). Differences in muscle strength and pain intensity did not reach significance (p= > .05). None of the included subjects reported to be limited due to pain in any of the measurements.

#### Multivariate analyses

The results of the multivariate analysis are provided in Table 2, in which the performance outcome is reported in panel 2a and the capacity measures in panel 2b and 2c.

**Table 2:** Multivariate regression models

**Panel 2a**	**Functional status (performance)**					
**Dependent**	**Predictor**	**B (SE)**	**ß**	**95% CI for B**		**p-value**
Total PAL	Age	-1.89(5.6)	-0.04	-13.10	9.31	p = .738
	Non-dancer vs dancer	37.71(29.3)	0.20	-20.68	96.11	p = .202
	BMI	4.06(5.6)	0.09	-7.29	15.41	p = .478
	GJH (Beighton ≥4)	-19.69(26.9)	-0.10	-73.33	33.94	p = .467
	Muscle Strength (N)	-0.04(0.5)	-0.16	-0.14	0.05	p = .373
	Pain intensity (mm)	-0.364(0.6)	-0.07	-1.66	0.05	p = .576
	Fatigue (CIS)	-0.03 (0.7)	-0.07	-1.47	1.40	p = .964
**Panel 2b**	**Functional status (capacity): Walking distance**					
**Dependent**	**Predictor**	**B (SE)**	**ß**	**95% CI for B**		**p-value**
6MWT^1^	Age	1.53(2.2)	0.05	-2.85	5.91	p = .488
	**Non-dancer vs dancer**	***81.5(11.5)***	***0.59***	***58.6***	***104.3***	***p = <.0001***
	**BMI**	***-8.4(2.2)***	***-0.28***	***-12.8***	***-3.9***	***p = <.0001***
	**GJH (Beighton ≥4)**	***-75.5(10.5)***	***-0.55***	***-96.5***	***-54.5***	***p = <.0001***
	**Muscle Strength (N)**	***0.04(0.02)***	***0.20***	***0.03***	***0.08***	***p = .034***
	Pain intensity (mm)	*0.48(0.25)*	*0.14*	*-0.29*	*0.98*	*p = .064*
	**Fatigue (CIS)**	***-0.58(0.28)***	***-0.18***	***-1.14***	***-0.02***	***p = .044***
	^*1*^*Regression equitation: 6MWT = 81.5(dance: 0/1) + -.8.4(BMI) + -.75.5(GJH: 0/1)) + .04(Muscle) + -.58(fatigue)*					
**Panel 2c**	**Functional status (capacity): Jumping capacity**					
**Dependent**	**Predictor**	**B (SE)**	**ß**	**95% CI for B**		**p-value**
SQH^2^	Age	.44(1.1)	0.04	-1.66	2.54	p = .678
	**Non-dancer vs dancer**	**18.1(5.5)**	**0.39**	**7.12**	**29.03**	**p = .002**
	BMI	-0.81(1.1)	-0.08	-2.94	1.32	p = .451
	**GJH (Beighton ≥4)**	**-11.2(5.1)**	**-0.24**	**-21.29**	**-1.16**	**p = .029**
	**Muscle Strength (N)**	**0.02(0.1)**	**0.36**	**0.01**	**0.04**	**p = .009**
	**Pain intensity (mm)**	**-0.26(0.1)**	**-0.23**	**-0.49**	**-0.02**	**p = .033**
	Fatigue (CIS)	-0.08(0.1)	-0.07	-0.35	0.19	p = .573
SH^3^	Age	1.14(1.1)	0.11	-0.95	3.24	p = .279
	**Non-dancer vs dancer**	**15.3(5.5)**	**0.34**	**4.35**	**26.17**	**p = .007**
	BMI	-1.49(1.1)	-0.17	-3.61	0.63	p = .165
	**GJH (Beighton ≥4)**	**-10.1(5.0)**	**-0.22**	**-20.19**	**-0.08**	**p = .048**
	Muscle Strength (N)	0.01(0.01)	0.06	-0.01	0.02	p = .650
	Pain intensity (mm)	-0.12(0.1)	-0.11	-0.36	0.12	p = .322
	Fatigue (CIS)	-0.14(0.1)	-0.14	-0.41	0.12	p = .289
	^*2*^*Regression equitation: SQH = 18.1(Dance: 0/1) +-11.2(GJH: 0/1) + .02(Muscle) + -.3(pain)*					
	^*3*^*Regression equitation: SH = 15.3(Dance: 0/1) +-10.1(GJH: 0/1)*					
	*Note: Total PAL: R*^*2*^*: .060, 6MWT: R*^*2*^ *= .678, SQH: R*^*2*^ *= .399, SH: R*^*2*^ *= .349*					

*Abbreviations:* ß (standardized beta), Body Mass Index (BMI), Generalized Joint Hypermobility (GJH), Total Physical Activity level (PAL), Metabolic Equivalent Tasks (METS), Six minute walk test (6MWT), Square Hop (SQH), Side hop (SH), Newton (N), Checklist Individual Strength (CIS) Significant associations are presented in bold.

#### Functional status: performance

Table 2 (panel 2a) illustrates the linear regression model concerning the association between functional status, expressed as total PAL (performance) and GJH, expressed in Beighton score of ≥4, controlled for confounders. The constructed model explained 6.0% of all variance, however none of the outcomes reached significance (p= > .05).

#### Functional status: capacity

Table 2 (panel 2b and 2c) illustrates the linear regression model concerning the association between functional status, expressed in 6MWT and jumping capacity (capacity) and GJH, expressed in Beighton score of ≥4, controlled for confounders.

In the first panel (6MWT) (Table 2b), the constructed model explained 67.8% of all variance. 6MWT was significantly negatively associated with GJH (B(SE):-75.5(10.5), p = <.0001), indicating lower scores on 6MWT associated with the presence of GJH. Furthermore, this association was negatively modified by BMI (B(SE):-8.4(2.2), p < .0001) and fatigue (B(SE):-.58(0.28), p = .044). Group (non-dancer vs dancer) (B(SE): 81.5(11.5), p = <.0001), and muscle strength (B(SE): .04(0.02), p = .034) were found to be positively associated with 6MWT, indicating dancers to have higher scores on 6MWT, and increased muscle strength. Age (p = .488) and pain intensity (p = .064) did not reach significance (p= > .05).

In the second panel on jumping capacity (SH, SQH) (Table 2c) similar results were found. When using SQH as dependent variable, 39.9% of all variance was explained by the model. SQH was significantly negatively associated with GJH (B(SE):-11.2(5.1), p = .029), indicating the presence of GJH to be associated with lower scores on SQH. In addition, pain intensity was negatively associated with SQH, indicating lower scores on SQH with increasing pain intensity level (B(SE):-0.26(0.1), p = .033). Dancing status was positively associated with SQH, with dancers scoring higher on SQH than non-dancers (B(SE): 18.1(5.5), p = .002). Muscle strength was found to be positively associated with SQH (B(SE): 0.02(0.1), p = .009). The remaining factors also failed to reach significance (p= > .05).

When using SH as the dependent variable, similar results were found. The model explained 34.9% of all variance. SH scores were found to be significantly negatively associated with the presence of GJH (B(SE):-10.1(5.0), p = .048), indicating lower score on SH when GJH was present. Again dancers showed higher scores on the SH in comparison to non-dancers (B(SE): 15.3(5.5), p = .007). The remaining factors did not reach significance (p= > .05).

## Discussion

Based on the presented data and after correction for confounders, GJH was associated with a decrease in functional status in terms of capacity, measured as walking distance and jumping capacity. No significant associations were found between GJH and a decrease in functional status in terms of performance (self-reported PAL). When regarding the capacity measures, both BMI and fatigue were found to be negative influencing factors on 6MWT. In contrast, increases in muscle strength and being a professional dancer were found to be positively associated with higher capacity of 6MWT and jump capacity.

The current sample consisted of young female adults recruited among healthcare students and a selected group of professional performing arts students (dancers). These two populations were found to differ on several clinical characteristics. First of all, dancers had significantly higher Beighton scores and lower scores on BMI, indicating that on average these subjects are slimmer and more flexible. An explanation for the observed difference can be found in the admission criteria for the professional dance education, where students with high flexibility and slim posture are more likely to be admitted. Despite the high flexibility and the significantly higher levels of fatigue of the included dancers, these individuals showed higher levels of physical capacity (6MWT, jumping capacity).

Besides the selection by flexibility and slender posture, dancers were admitted to the education when they excel in performing complex choreographs. We postulate that as result of the stringent selection criteria and training for professional dancers, they were able to perform better on all capacity measures, and especially on the jumping tests that also rely heavily on general gross motor competences. Previous studies on children and adults diagnosed with symptomatic forms of hypermobility have been found to have less motor control [14] and proprioception. Combined with a loss in physical fitness in terms of muscle strength and cardiovascular exercise capacity [15,30], this may lead to functional decline in individuals with symptomatic hypermobility. However, in the current study the presence of GJH was independently associated with a lower physical capacity (both 6MWT and jumping capacity), even in physically well-trained professional dancers. This could imply that connective tissue laxity, in terms of GJH, is a risk factor for functional decline rather than the consequence of deconditioning [39].

From the current data we can conclude that in females, muscle strength is a promoting factor in regard to functional status in terms of 6MWT, and furthermore, ascribes the potential benefit of strength training for improved capacity. However, due to the design of the study, the current study lacks methodological strength to prove such an assumption. Besides muscle weakness, reduced cardiovascular exercise tolerance has also been reported in literature in both symptomatic [30] and non-symptomatic GJH [11]. This was not included in the current analysis, however most functional activities do not require maximum exercise tolerance like effort, and even more so, most individuals rarely engage in sustained maximum cardiovascular efforts. When looking at the levels of muscle strength and functional capacity in dancers classified with GJH, these were considerably higher in comparison to non-dancers with GJH. Higher levels of physical fitness and motor competence could explain this difference and may be the result of training which is in line with current literature [40].

Pain intensity was not found to be a factor of influence on 6MWT. This could be related to the low intensity and stable, cyclic nature of walking like in the 6MWT. Pain intensity, which was a negatively influencing factor on SQH, might have been a more important factor in more dynamic activities like SQH, exerting more forces on joint surfaces, especially in movements requiring axial rotational forces.

According to the multivariate analyses, GJH had a negative contribution in all capacity variables. The presence of GJH, which could be the result of abnormal connective tissue laxity, may result in higher demands on active joint stabilization mechanisms [41,42]. In combination with proprioceptive inaccuracy, during highly coordinative tasks like walking, demanding adaptive strategies like co-contraction or prolonged activation of certain muscle groups are required to stabilize joints during these activities [43]. Further, higher energy demands may be required, which could lead to fatigue [44]. In our data next to GJH, fatigue was also a negative contributor to the walking distance supporting this theory.

We found a profound difference between performance and capacity, which could be explained by the use of questionnaires which are based on subjective experience and recall. The lack of association in performance could be the result of estimation bias due to recall. In addition capacity measures are only able to reflect activity impairments partially as they do not account for environmental factors. The presence of recall bias could be avoided, while accounting for environmental factors, by the use of objective performance measures like 3d accelerometry. Although not possible in the current study due to time constraints, this would be recommended for future studies.

When interpreting the presented results, the following limitations should be considered. Firstly, the included population was selected from healthcare students, who may attend more to issues of healthy living when compared to the general population, as well as professional dance students, who have highly demanding physical routines in their daily life. In addition, the current included population was limited to only females due to the lack of available male dancers at time of inclusion. This limits the generalizability of these results to the general population. Still, these results do illustrate that biomechanical factors like GJH have direct consequences for functional status, however the magnitude of that effect could differ between populations, with this group being a high risk population. Secondly, the used outcomes of functional status are not dance specific, due to the issues of comparability between dancers and non-dancers. The effect of GJH on dancing itself might differ and should be investigated. Still, both walking and jumping are also applicable to dancers and could indicate that also GJH can have profound effects on dancing and might even be larger due to the high demands on joint stability during dancing. Currently the use of the Beighton score in order to determine GJH is highly debated. Although the Beighton score is still considered to be the “gold standard” for classifying GJH, aspects of its operationalization and the validity of the cut-off values are still in need of further research and a revision of the way GJH is determined is needed [40]. Thirdly, environmental and psychological factors were not incorporated in the current study. Social status, peer pressure, kinesiophobia and (pain) coping strategies could explain the discrepancies between the performance and capacity measures of functional status [45]. However, this is beyond the scope of the current paper but could prove to be a vital area of future research in order to fully understand the interaction between functional status and the human organism in its own individual context. Such knowledge is critical in order to develop effective treatment modalities that are able to diminish activity impairments for subjects diagnosed with symptomatic forms of GJH. It may also be important for treatment of other musculoskeletal diseases. Finally, based on the study design these results offer no causative evidence, therefore, these observations need to be replicated in longitudinal observational studies incorporating individuals with both symptomatic and non-symptomatic GJH.

## Conclusion

We conclude, that GJH in females was associated with a decrease in functional status in terms of capacity, measured as walking distance and jumping capacity.

GJH is a factor to consider when assessing functional status in terms of walking and jumping capacity, possibly due to altered joint biomechanics, compromised by structural integrity of connective tissue. Incorporating the assessment of musculoskeletal complaints (pain), fatigue, and muscle strength could provide additional clinically relevant information. However, the role of connective tissue laxity, expressed by GJH, for functional status is still poorly understood.

## Abbreviations

GJH: Generalized joint Hypermobility; BMI: Body Mass Index; PAL: Total Physical Activity level; METS: Metabolic Equivalent Tasks; 6MWT: Six minute walk test; SQH: Square Hop; SH: Side Hop; N: Newton; CIS: Checklist Individual Strength.

## Competing interests

The authors declare that they have no competing interests.

## Authors’ contributions

MCS was involved in the conception of the design, data collection, data analysis, drafted the manuscript. JEV was involved in data collection, data analysis and assisted in drafting the manuscript. BJK assisted in the data analysis and the drafting of the manuscript. FN was involved in the drafting of the manuscript and the conception of the design, RHHE was the coordinating researcher and was involved in the conception of the design and the drafting of the manuscript. All authors read and approved the final manuscript.

## Pre-publication history

The pre-publication history for this paper can be accessed here:

http://www.biomedcentral.com/1471-2474/15/243/prepub
